# Antenatal Dexamethasone Exposure in Preterm Infants Is Associated with Allergic Diseases and the Mental Development Index in Children

**DOI:** 10.3390/ijerph13121206

**Published:** 2016-12-03

**Authors:** Wan-Ning Tseng, Chih-Cheng Chen, Hong-Ren Yu, Li-Tung Huang, Ho-Chang Kuo

**Affiliations:** 1 Department of Pediatrics, Kaohsiung Chang Gung Memorial Hospital, Kaohsiung 83301, Taiwan; proteus@cgmh.org.tw (W.-N.T.); charllysc@cgmh.org.tw (C.-C.C.); yuu2004taiwan@yahoo.com.tw (H.-R.Y.); 2 Chang Gung University College of Medicine, Kaohsiung 83301, Taiwan; 3 Department of Traditional Chinese Medicine, Chang Gung University, Taoyuan 33302, Taiwan

**Keywords:** prenatal, dexamethasone, mental development index (MDI), psychomotor developmental index (PDI), allergy

## Abstract

**Background:** Antenatal steroid administration may benefit fetal lung maturity in preterm infants. Although some studies have shown that this treatment may increase asthma in childhood, the correlation between antenatal dexamethasone exposure and allergic diseases remains unclear. The purpose of this study is to investigate the association between antenatal dexamethasone and T cell expression in childhood allergic diseases. **Methods:** We recruited a cohort of preterm infants born at Kaohsiung Chang Gung Memorial Hospital between 2007 and 2010 with a gestational age of less than 35 weeks and body weight at birth of less than 1500 g. The status of antenatal exposure to steroids and allergic diseases were surveyed using a modified ISAAC questionnaire for subjects aged 2–5 years old. We analyzed Th1/Th2/Th17 expression of mRNA, cytokines (using the Magpix^®^ my-system), and mental development index (MDI). **Results:** Among the 40 patients that were followed, the data showed that the antenatal dexamethasone exposure group (*N* = 24) had a significantly higher incidence of allergic diseases (75.0% vs. 18.8%, *p* < 0.0001) when compared to the non-dexamethasone exposure group (*N* = 16), especially with regard to asthma (41.7% vs. 0.0%, *p* = 0.003) and allergic rhinitis (58.3% vs. 18.8%, *p* = 0.013), but not atopic dermatitis. No statistical difference was observed in the mRNA expression levels of total white blood cell count between the dexamethasone exposure and non-exposure groups (*p* > 0.05). However, the asthma group had higher IL-5 levels (*p* = 0.009), and the MDI was shown to be significantly higher in the dexamethasone exposure group (90.38 ± 3.31 vs. 79.94 ± 3.58, *p* = 0.043) while no significant difference was found between the PDI of the two groups. **Conclusions:** Exposure to antenatal dexamethasone in preterm infants will increase their susceptibility to allergic diseases, particularly asthma and allergic rhinitis. Preterm infants’ exposure to antenatal dexamethasone also results in higher MDI scores. Such increases in allergic diseases may be related to increased IL-5 and IL-10 levels.

## 1. Introduction

In recent years, the prevalence of such allergic diseases as atopic dermatitis, asthma, and allergic rhinitis has increased worldwide, but especially in developed countries [1,2,3,4]. Such increase has been attributed to various environmental changes, including hygiene factors, increased use of antimicrobial agents, consumption of sterile foods, and reduced family size, all of which result in lower infection rates during childhood and reduced early contact with microbes. These factors have profoundly impacted immune system function and increased the development of allergic disease [5,6,7,8]. By interfering with the development of a child’s immune system, this situation tends to be directed towards the T-helper (Th) 2 immune response [9,10,11]. Experimental and epidemiologic data have indicated that early microbial exposure is critical for Th1-skewed immune responses in healthy children during the postnatal period [12].

The perinatal period is particularly sensitive to various risks that can permanently alter stress-regulating systems and cause neuropsychopathologies to ensue [13,14]. Unlike endogenous glucocorticoids, synthetic glucocorticoids bind mainly to the glucocorticoid receptor because the mineralocorticoid receptor has a low affinity for synthetic glucocorticoids. Synthetic glucocorticoids (betamethasone/dexamethasone) are administered to pregnant women who are at risk of preterm delivery in order to promote fetal maturation and reduce neonatal morbidity and mortality. However, in human pregnancy, evidence has indicated that fetal exposure to synthetic glucocorticoids can have harmful effects on birth outcome, childhood cognition, long-term behavior, and allergic diseases (mainly asthma) [15]. Pole et al. reported that antenatal steroid therapy appears to be an independent risk factor for the development of asthma in children between the ages of 3–6 years old [15]. Furthermore, Dietert et al. showed that fetal exposure to dexamethasone (even in the lowest doses tested) results in marked and persistent functional loss of delayed type hypersensitivity (DTH, a Th1 immune response), which was not found in exposed adults [16].

Administering antenatal corticosteroids to women at risk of preterm delivery to promote fetal lung maturation and improve prognosis has been established as standard care these days [17,18,19]. However, such treatment may possibly cause adverse effects, such as reduced fetal growth [20,21] and epilepsy [19]. In recent years, some studies have pointed out that this intervention may be correlated with increasing asthma during childhood [15,22], but the method with which it does so is still unknown. After performing a literature review, we found that no studies addressed antenatal dexamethasone exposure in preterm infants and allergic diseases, such as allergic rhinitis and atopic dermatitis.

Hydrocortisone-treated infants were at a lower risk of having a Mental Development Index (MDI) of <70, as reported by Watterberg et al. [23] in the outcomes of extremely low birth weight infants at a corrected age of 18 to 22 months. However, Wilson-Costello et al. reported that each 1 mg/kg dose of steroid treatment was associated with a 2.0-point reduction on the MDI [24]. Therefore, this study was conducted to investigate the relationship among antenatal dexamethasone exposure, childhood allergic disease, and mental development. Furthermore, the association between antenatal dexamethasone exposure and T helper cell expression (including Th1/Th2/Th17/Treg) is explored to better understand the possible mechanism.

## 2. Material and Methods

We recruited a cohort of preterm infants born at Kaohsiung Chang Gung Memorial Hospital with a gestational age of less than 35 weeks and body weight at birth less than 1500 grams in the years 2007–2010. Antenatal steroids dosage and courses were a single course defined as two 12 mg doses of betamethasone given 24 h apart or four 6 mg doses of dexamethasone given 12 h apart. Exposure to antenatal steroids was recorded pursuant to maternal medical records. Questionnaires related to allergic diseases (asthma, allergic rhinitis, and atopic dermatitis) were modified from the International Study of Asthma and Allergies in Childhood (ISAAC) [25] and distributed to the parents of children when they were 2 and 5 years old.

We also performed comprehensive neurodevelopmental evaluations of the Bayley Scale score II of the Mental Development Index (MDI) and Psychomotor Developmental Index (PDI) of the children at age 2 and 5 years and associated them with dexamethasone exposure status. This study has been approved by the Institutional Review Board (IRB No. 102-2895B) of Chang Gung Memorial Hospital. The IRB approved this consensual procedure. We only collected blood samples after obtaining written informed consent from the children’s guardians or parents. The participants’ consent was recorded with a decoded method.

### 2.1. Measurement of Plasma Cytokine Levels Associated with Th Cell Subset Immunity Using the Magpix^®^ My-System

We measured the plasma levels of cytokines with the Magpix^®^ my-system (Merck Millipore; Kenilworth, NJ, USA). Plasma concentrations of interleukin (IL)-2, interferon-γ (IFN-γ), IL-4, IL-5, IL-13, interferon gamma inducible protein-10 (IP-10), and IL-17A were assessed using the Milliplex Assay system (Merck Millipore). We used a study method that was modified from our previous reports [26,27]. First, primary antibody conjugated beads were incubated with diluted standards or plasma from human subjects overnight and then with detector antibodies for 1 h at room temperature. Fluorescent detection was performed and incubated for 30 min with fluorescent dye-conjugated streptavidin-phycoerythrin. Cytokine levels were measured using the Magpix^®^ my-system and analyzed with Milliplex^®^ analyst 5.1 software (Merck Millipore).

### 2.2. Real-Time Quantitative RT-PCR Analysis of Th Cell-Related mRNA Expression

We isolated the total RNA from white blood cells with a FavorPrep Blood/Cultured Cell Total RNA Purification kit (catalog no. FABRK001-1; Favorgen Biotech, Pingtung City, Taiwan). Reverse-transcription was performed using the High Performance Reverse Transcriptase System (EPICENTRE). The expression levels of T-bet, Gata3, and RORγt were detected with real-time RT-PCR using SYBR Green PCR Master Mix and ABI Prism 7500 Sequence Detection System (Applied Biosystems, Foster City, CA, USA). The T-bet, Gata3, and RORγt expression levels were normalized using 18S rRNA as an internal control and were presented as absolute expression levels. The primers used for amplifying 18S rRNA were 5′-GTA ACC CGT TGA ACC CCA TT-3′ (forward), 5′-CCA TCC AAT CGG TAG CG-3′ (reverse). The primers used for T-bet mRNA were 5′-AAC ACA GGA GCG CAC TGG AT-3′ (forward) and 5′-TCT GGC TCT CCG TCG TTC A-3′ (reverse); for GATA-3 mRNA: 5′-GTG CTT TTT AAC ATC GAC GGT C-3′ (forward) and 5′-AGG GGC TGA GAT TCC AGG G-3′ (reverse), and those for RORγt mRNA were 5′-TTT TCC GAG GAT GAG ATT GC-3′ (forward) and 5′-CTT TCC ACA TGC TGG CTA CA-3′ (reverse).

### 2.3. Statistical Analysis

Cytokine levels, mRNA expression levels, MDI, and PDI between the two groups were tested using the Student’s *t-*test and/or the Mann–Whitney *U* test. A *p*-value <0.05 was considered statistically significant. All statistical tests were performed using SPSS 14.0 for Windows (SPSS, Inc., Chicago, IL, USA).

## 3. Results

### 3.1. Dexamethasone Exposure Group Had a Higher Incidence of Allergic Diseases

We enrolled a total of 40 patients during the study period, 24 (60.0%) of which had been exposed to antenatal dexamethasone (exposure group); the other 16 patients (40.0%) had not been exposed to antenatal dexamethasone (non-exposure group). As shown in Table 1, no significant differences were found in gender distribution or gestational age (*p* > 0.05), but we observed a significant difference in the exposure group’s higher birth body weight (1210.83 ± 43.06 gm vs. 1010.00 ± 44.13 gm, *p* = 0.003). This study consisted of 21 males and 19 females, with no significant difference regarding their body weight at birth (*p* = 0.75).

The exposure group was found to have a significantly higher incidence of allergic diseases between the ages of 2–5 years old (75.0% vs. 18.8%, *p* < 0.0001) when compared to the non-exposure group. Of the allergic diseases, asthma (41.7% vs. 0.0%, *p* = 0.003) and allergic rhinitis (58.3% vs. 18.8%, *p* = 0.013) were found to increase after exposure to antenatal dexamethasone; however, the same was not found with the incidence of atopic dermatitis (25.0% vs. 6.3%, *p* = 0.126).

### 3.2. Higher Plasma Levels of IL-5 and IL-10 Were Found in the Asthma and Allergic Rhinitis Group

The concentrations of Th1 cytokines (IFNγ, IL-2, and IP-10) did not differ significantly between the groups with or without exposure to antenatal dexamethasone. The concentrations of Th2 cytokines (IL-5 and IL-13) were relative higher in the dexamethasone exposure group but did not reach statistical significance (Table 2). Patients with asthma had significantly higher plasma levels of IL-5 (0.92 ± 0.22 vs. 0.45 ± 0.07 pg/mL, *p* = 0.009) and IL-10 (2.13 ± 0.32 vs. 1.88 ± 0.56 pg/mL, *p* = 0.026), and patients with allergic rhinitis also showed elevated IL-5 levels (0.77 ± 0.17 vs. 0.42 ± 0.04 pg/mL, *p* = 0.026) and IL-10 concentrations (3.14 ± 0.91 vs. 1.05 ± 0.16, *p* = 0.013). Regarding patients with atopic dermatitis, IP-10 was significantly higher (320.86 ± 52.84 vs. 211.74 ± 15.17 pg/mL, *p* = 0.010), but concentrations of Th2 cytokines (IL-5, and IL-13) did not reach statistical significance.

When studying the gene expression, we found no obvious differences between the groups with or without antenatal dexamethasone exposure with regard to T-bet, GATA-3, Foxp-3, and RORγt (Table 3).

### 3.3. Dexamethasone Exposure Group Had a Higher MDI Score

**Figure 1:**
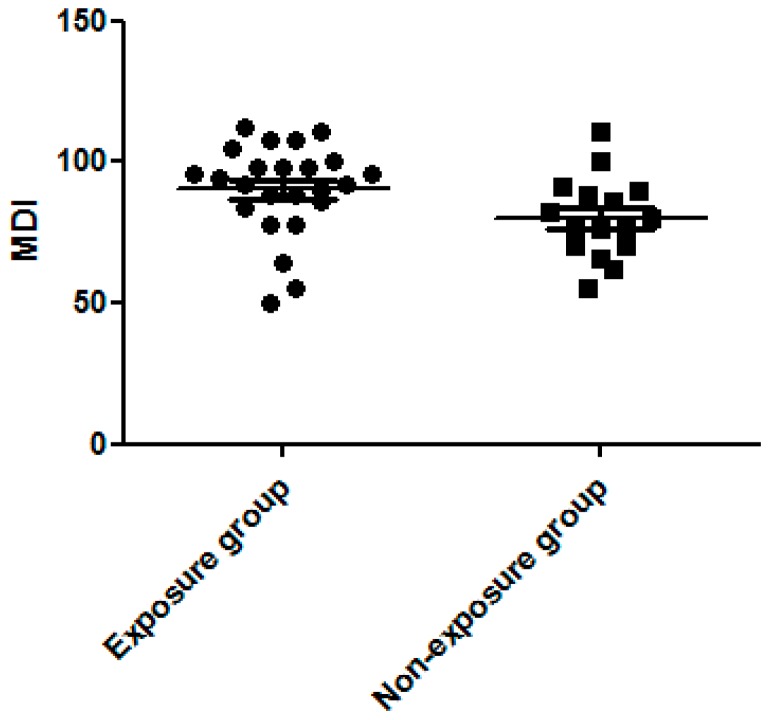
Significantly higher Mental Development Index (MDI) in the dexamethasone exposure group than the non-exposure group.

**Figure 2:**
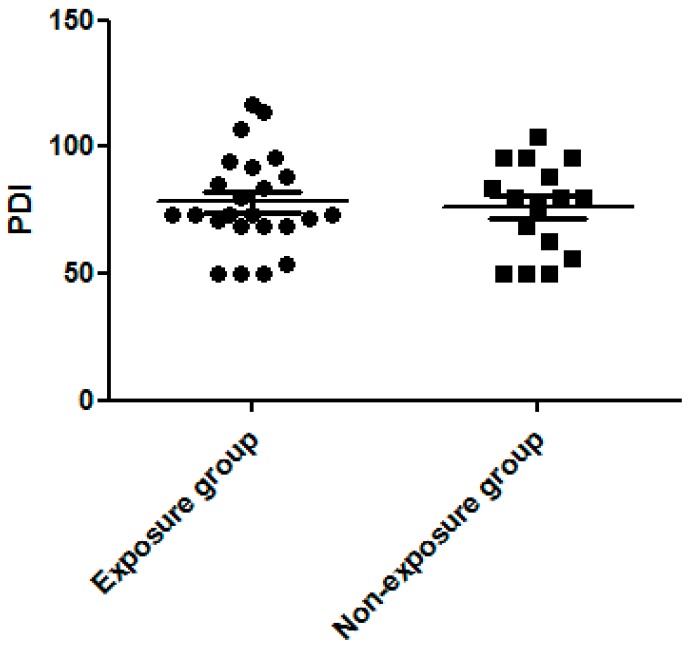
No significant difference in the Psychomotor Developmental Index (PDI) between the dexamethasone exposure and non-exposure groups.

We found MDI to be significantly higher in the dexamethasone exposure group than the non-exposure group (90.38 ± 3.31 vs. 79.94 ± 3.58, *p* = 0.043, Figure 1). The PDI score did not differ significantly between the two groups (78.17 ± 3.81 vs. 76.13 ± 4.51, *p* > 0.05, Figure 2). The percentage of MDI < 70 was also lower in the exposure group than in the non-exposure group (12.5% vs. 18.8%, *p* > 0.05) but did not reach statistical significance.

## 4. Discussion

Administering antenatal steroids has become an important intervention method for preventing bronchopulmonary dysplasia in premature infants. However, it may also affect other aspects, such as fetal growth [20,21], kidneys [28], cardiovascular adaption [29], and the immune system. In this study, we found antenatal steroid exposure to be correlated with an increased incidence of allergic diseases, particularly asthma and allergic rhinitis. These findings agree with a previous study by Pole et al. that reported that antenatal steroid therapy appears to be a risk factor for asthma among children between the ages of 36 and 72 months [15]. The etiology of pediatric asthma includes various exposures and complex interactions. In infants, such factors include low birth body weight, gestational age, measurements at birth (body length and head circumference), malposition, and threatened labor. Maternal risk factors include maternal asthma history, smoking during pregnancy, mode of delivery, maternal age, prematurity, and breastfeeding status. Annesi-Maesano et al. reported that threatened labor could result in a significantly increased risk of asthma for the child [30]. In Taiwan, mothers with threatened preterm labor before 34 weeks are routinely given corticosteroid to promote fetal lung maturation and prevent hyaline membrane disease in accordance with the American College of Obstetricians and Gynecologists’ Committee on Obstetric Practice (ACOG) guidelines [31]. While the precise mechanisms are still unclear, the priming of the Th1/Th2 system has been reported to occur during late gestation, as well as that pulmonary systems are already programmed during intrauterine life. Th1 immunity becomes markedly diminished during pregnancy, which results in maternal tolerance of the antigens derived from the paternal chromosomes. Diminished Th1 activity during pregnancy may help explain why pregnant women have a higher incidence of infections than non-pregnant women, especially with intracellular pathogens. The Th2 cytokines (IL-5) and regulatory T cytokine (Treg, IL-10) were also elevated in patients with asthma and allergic rhinitis but not atopic dermatitis in this study. The plasma levels of IP-10 were higher in atopic dermatitis, which suggests a different immune reaction between atopic dermatitis and asthma/allergic rhinitis. The modulation of the immune system may occur before 2 years old, prior to developing the allergic disease. Any allergic process may be the result of the fetus’s exposure to the mediators in utero. Whether corticosteroid therapy in the late gestation is directly associated with childhood asthma or whether it is a mediating factor of threatened labor still requires further study, but it seems to be a risk factor for childhood asthma. We first reported the increased incidence of allergic rhinitis in children with antenatal steroid exposure in this study. Dietert et al. showed that fetal exposure to dexamethasone (even at its lowest doses) provokes marked and persistent functional loss of DTH (Th1 immune response) [16]. Other studies have indicated that inflammation in atopic dermatitis is biphasic: Th2 cells in acute atopic dermatitis; Th1 cells in chronic atopic dermatitis. This phenomenon may explain why antenatal steroids exposure in preterm infants is correlated with asthma and allergic rhinitis, which are considered Th2 cell-associated inflammatory diseases, while atopic dermatitis is not. In this study, we found no significant differences in mRNA of T-bet, RORγt, or cytokines (IL-17A, IFNγ, IL-2, and IP-10) between the prenatal exposure and non-exposure groups. This study also demonstrated no significant differences in Th2 immune response (GATA-3, IL-5, and IL-13) and Treg immune response (Foxp-3 and IL-10). A limitation of this study is the number of cases examined. Furthermore, no studies have yet discussed antenatal steroid exposure or change in cytokines from birth to 2 years old. Therefore, we will consider designing another study that focuses on this group. The impact of prenatal dexamethasone exposure on Th1/Th2/Th17 and Treg immune response needs further evaluation before a definite conclusion can be reached.

Our study also found a significantly higher MDI in the dexamethasone exposure group, but no significant differences in the PDI score between the two groups. We used the Mental Developmental Index to measure language, memory, and problem-solving abilities. Alexander et al. noted the long-lasting effects of antenatal synthetic corticosteroids exposure on hypothalamic-pituitary-adrenal reactivity in term infants, which may have significant implications with regard to the risk for stress-related physical and psychiatric disorders [32]. This may explain the correlation between the use of antenatal corticosteroids and mental development. However, Asztalos et al. demonstrated that, in preterm infants, the neurodevelopmental outcome remained the primary factor (such as gestational age at birth), which was contributory regardless of the number of courses of antenatal synthetic corticosteroid therapy [33]. Dexamethasone is not only used to promote fetus lung maturation but also to prevent further intraventricular hemorrhaging in preterm births before 34 weeks’ gestation [34]. For preterm infants, the benefit offered by dexamethasone may exceed its disadvantages. However, this issue requires further study due to the limitations of our case numbers. The Psychomotor Developmental Index generally measures motor skills, but this was not related to dexamethasone exposure in our study.

## 5. Conclusions

Exposure to antenatal dexamethasone in preterm infants may increase susceptibility to allergic diseases (especially with regard to asthma and allergic rhinitis) and result in higher MDI scores. Although the possible mechanisms for this action are still unknown, they may be related to increased IL-5 and IL-10 levels.

## Figures and Tables

**Table 1:** Demographic data of the patients with or without antenatal dexamethasone exposure.

Demographic Data	Exposure Group (*n* = 24)	Non-Exposure Group (*n* = 16)	*p*-Value
Male gender	13 (54.2%)	8 (50.0%)	0.796
Gestational age (week)	29.63 ± 0.50	28.06 ± 0.73	0.074
Birth body weight (gm)	1210.83 ± 43.06	1010.00 ± 44.13	0.003 *
Age (years)	3.89 ± 0.14	4.17 ± 0.20	0.242
Allergic disease	18 (75.0%)	3 (18.8%)	<0.0001 *
Asthma	10 (41.7%)	0 (0.0%)	0.003 *
Allergic rhinitis	14 (58.3%)	3 (18.8%)	0.013 *
Atopic dermatitis	6 (25.0%)	1 (6.3%)	0.126

Data are presented as percentage and mean with standard error. * *p* < 0.05. *p*-Values were determined using the Student’s *t*-test.

**Table 2:** The plasma levels of cytokines from patients with or without prenatal dexamethasone exposure.

Cytokines	Exposure Group (*n* = 24)	Non-Exposure Group (*n* = 16)	*p*-Value
Interferon-gamma (pg/mL)	3.20 ± 0.75	3.39 ± 0.65	0.856
IL-2 (pg/mL)	1.16 ± 0.22	0.68 ± 0.09	0.093
IL-5 (pg/mL)	0.67 ± 0.12	0.42 ± 0.06	0.137
IL-10 (pg/mL)	2.44 ± 0.67	1.19 ± 0.27	0.148
IL-13 (pg/mL)	0.34 ± 0.24	0.20 ± 0.13	0.647
IL-17A (pg/mL)	2.91 ± 1.08	2.01 ± 0.34	0.508
IP-10 (pg/mL)	240.46 ± 21.66	216.41 ± 26.19	0.485

IL: interleukin; IP-10: interferon gamma inducible protein-10. Data are presented as mean with standard error. * *p* < 0.05. *p*-Values were tested using the Student’s *t*-test.

**Table 3:** The mRNA expression of T helper cells from patients with or without prenatal dexamethasone exposure.

mRNA	Exposure Group (*n* = 24)	Non-Exposure Group (*n* = 16)	*p*-Value
T-bet	0.74 ± 0.18	0.81 ± 0.36	0.857
GATA-3	0.74 ± 0.13	0.97 ± 0.22	0.322
Foxp-3	0.86 ± 0.12	0.96 ± 0.16	0.600
RORγt	0.83 ± 0.17	1.01 ± 0.25	0.537

Data are presented as mean with standard error. * *p* < 0.05. *p*-Values were tested with the Student’s *t*-test.

## References

[1] Liu C.A., Wang C.L., Chuang H., Ou C.Y., Hsu T.Y., Yang K.D. (2003). Prenatal prediction of infant atopy by maternal but not paternal total IgE levels. J. Allergy Clin. Immunol..

[2] Eder W., Ege M.J., von Mutius E. (2006). The asthma epidemic. N. Engl. J. Med..

[3] Ronmark E., Bjerg A., Perzanowski M., Platts-Mills T., Lundback B. (2009). Major increase in allergic sensitization in schoolchildren from 1996 to 2006 in northern Sweden. J. Allergy Clin. Immunol..

[4] Bisgaard H., Simpson A., Palmer C.N., Bonnelykke K., McLean I., Mukhopadhyay S., Pipper C.B., Halkjaer L.B., Lipworth B., Hankinson J. (2008). Gene-environment interaction in the onset of eczema in infancy: Filaggrin loss-of-function mutations enhanced by neonatal cat exposure. PLoS Med..

[5] Chang T.W., Pan A.Y. (2008). Cumulative environmental changes, skewed antigen exposure, and the increase of allergy. Adv. Immunol..

[6] Putney J.W. (2009). Capacitative calcium entry: From concept to molecules. Immunol. Rev..

[7] Furukawa S., Matsubara T., Motohashi T., Sasai K., Nakachi S., Umezawa Y., Yabuta K. (1991). Increased expression of Fc epsilon R2/CD23 on peripheral blood B lymphocytes and serum IgE levels in Kawasaki disease. Int. Arch. Allergy Appl. Immunol..

[8] Anderson W.J., Watson L. (2001). Asthma and the hygiene hypothesis. N. Engl. J. Med..

[9] Riedler J., Braun-Fahrlander C., Eder W., Schreuer M., Waser M., Maisch S., Carr D., Schierl R., Nowak D., von Mutius E. (2001). Exposure to farming in early life and development of asthma and allergy: A cross-sectional survey. Lancet.

[10] Cullinan P., Harris J.M., Newman Taylor A.J., Jones M., Taylor P., Dave J.R., Mills P., Moffat S.A., White C.W., Figg J.K. (2003). Can early infection explain the sibling effect in adult atopy?. Eur. Respir. J..

[11] Bach J.F. (2002). The effect of infections on susceptibility to autoimmune and allergic diseases. N. Engl. J. Med..

[12] Yang K.D., Ou C.Y., Hsu T.Y., Chang J.C., Chuang H., Liu C.A., Liang H.M., Kuo H.C., Chen R.F., Huang E.Y. (2007). Interaction of maternal atopy, CTLA-4 gene polymorphism and gender on antenatal immunoglobulin E production. Clin. Exp. Allergy.

[13] Huang L.T. (2011). The link between perinatal glucocorticoids exposure and psychiatric disorders. Pediatr. Res..

[14] Huang L.T. (2011). Perinatal programming of neuropsychiatric disorders. J. Formos Med. Assoc..

[15] Pole J.D., Mustard C.A., To T., Beyene J., Allen A.C. (2009). Antenatal steroid therapy for fetal lung maturation: Is there an association with childhood asthma?. J. Asthma.

[16] Dietert R.R., Lee J.E., Olsen J., Fitch K., Marsh J.A. (2003). Developmental immunotoxicity of dexamethasone: Comparison of fetal versus adult exposures. Toxicology.

[17] Martin R.J., Fanaroff A.A. (2013). The preterm lung and airway: Past, present, and future. Pediatr. Neonatol..

[18] Wong D., Abdel-Latif M., Kent A., NICUS Network (2014). Antenatal steroid exposure and outcomes of very premature infants: A regional cohort study. Arch. Dis. Child. Fetal Neonatal Ed..

[19] Eriksson L., Haglund B., Ewald U., Odlind V., Kieler H. (2009). Short and long-term effects of antenatal corticosteroids assessed in a cohort of 7827 children born preterm. Acta Obstet. Gynecol. Scand..

[20] Braun T., Husar A., Challis J.R., Dudenhausen J.W., Henrich W., Plagemann A., Sloboda D.M. (2013). Growth restricting effects of a single course of antenatal betamethasone treatment and the role of human placental lactogen. Placenta.

[21] Murphy K.E., Willan A.R., Hannah M.E., Ohlsson A., Kelly E.N., Matthews S.G., Saigal S., Asztalos E., Ross S., Delisle M.F. (2012). Effect of antenatal corticosteroids on fetal growth and gestational age at birth. Obstet. Gynecol..

[22] Pole J.D., Mustard C.A., To T., Beyene J., Allen A.C. (2008). Antenatal steroid therapy and childhood asthma: Is there a possible link?. Med. Hypotheses.

[23] Watterberg K.L., Shaffer M.L., Mishefske M.J., Leach C.L., Mammel M.C., Couser R.J., Abbasi S., Cole C.H., Aucott S.W., Thilo E.H. (2007). Growth and neurodevelopmental outcomes after early low-dose hydrocortisone treatment in extremely low birth weight infants. Pediatrics.

[24] Wilson-Costello D., Walsh M.C., Langer J.C., Guillet R., Laptook A.R., Stoll B.J., Shankaran S., Finer N.N., van Meurs K.P., Engle W.A. (2009). Impact of postnatal corticosteroid use on neurodevelopment at 18 to 22 months’ adjusted age: Effects of dose, timing, and risk of bronchopulmonary dysplasia in extremely low birth weight infants. Pediatrics.

[25] Tsakok T., Weinmayr G., Jaensch A., Strachan D.P., Williams H.C., Flohr C., ISAAC Phase 2 Study Group (2015). Eczema and indoor environment: Lessons from the International Study of Asthma and Allergies in Childhood (ISAAC) Phase 2. Lancet.

[26] Kuo H.C., Wang C.L., Liang C.D., Yu H.R., Huang C.F., Wang L., Hwang K.P., Yang K.D. (2009). Association of lower eosinophil-related T helper 2 (Th2) cytokines with coronary artery lesions in Kawasaki disease. Pediatr. Allergy Immunol..

[27] Huang Y., Shan J., Zhang C., Zhang J., Feng L., Li S., Li Y. (2010). Peripheral blood T regulatory cell counts may not predict transplant rejection. BMC Immunol..

[28] Cattarelli D., Chirico G., Simeoni U. (2002). Renal effects of antenatally or postnatally administered steroids. Pediatr. Med. Chir..

[29] Morrison J.L., Botting K.J., Soo P.S., McGillick E.V., Hiscock J., Zhang S., McMillen I.C., Orgeig S. (2012). Antenatal steroids and the IUGR fetus: Are exposure and physiological effects on the lung and cardiovascular system the same as in normally grown fetuses?. J. Pregnancy.

[30] Annesi-Maesano I., Moreau D., Strachan D. (2001). In utero and perinatal complications preceding asthma. Allergy.

[31] Committee on Obstetric Practice (2002). ACOG committee opnion: Antenatal corticosteroid therapy for fetal maturation. Obstet. Gynecol..

[32] Alexander N., Rosenlocher F., Stalder T., Linke J., Distler W., Morgner J., Kirschbaum C. (2012). Impact of antenatal synthetic glucocorticoid exposure on endocrine stress reactivity in term-born children. J. Clin. Endocrinol. Metab..

[33] Asztalos E., Willan A., Murphy K., Matthews S., Ohlsson A., Saigal S., Armson A., Kelly E., Delisle M.F., Gafni A. (2014). Association between gestational age at birth, antenatal corticosteroids, and outcomes at 5 years: Multiple courses of antenatal corticosteroids for preterm birth study at 5 years of age (MACS-5). BMC Pregnancy Childbirth.

[34] Miracle X., Di Renzo G.C., Stark A., Fanaroff A., Carbonell-Estrany X., Saling E., Coordinators of World Associatin of Perinatal Medicine Prematurity Working Group (2008). Guideline for the use of antenatal corticosteroids for fetal maturation. J. Perinat. Med..

